# Picosecond pulsed electric fields induce apoptosis in a cervical cancer xenograft

**DOI:** 10.3892/mmr.2014.2953

**Published:** 2014-11-14

**Authors:** JIA JIA, ZHENG-AI XIONG, QIN QIN, CHEN-GUO YAO, XIAO-ZHEN ZHAO

**Affiliations:** 1 Department of Obstetrics and Gynecology, The Second Affiliated Hospital of Chongqing Medical University, Chongqing 400010, P.R. China; 2 State Key Laboratory of Power Transmission Equipment and System Security and New Technology, Chongqing University, Chongqing 400044, P.R. China

**Keywords:** picosecond pulsed electric fields, human cervical carcinoma, nude mouse xenograft, apoptosis, caspase

## Abstract

The aim of the present study was to evaluate the efficacy of picosecond pulsed electric fields (psPEF) on a cervical cancer xenograft. Human cervical cancer xenografts were established in nude mice by transplantation of HeLa cells, and the tumors were then treated with psPEF. The histological changes were observed by hematoxylin-eosin staining and transmission electron microscopy. The rate of tumor cell apoptosis was determined using a terminal deoxynucleotidyl-transferase-mediated dUTP nick end labeling assay. The mitochondrial transmembrane potential of the tumor cells was detected by laser scanning confocal microscopy, and the activity of caspase-3, -8, -9 and -12 was determined. The inhibitory rate seven days post-psPEF treatment was also calculated. The results showed that exposure to psPEF led to an increased rate of apoptosis, collapse of mitochondrial transmembrane potential, and activation of caspases. The inhibitory rate was 9.11% at day 7. The results of the present study indicate that psPEF may induce apoptosis in a cervical cancer xenograft through the endoplasmic reticulum stress and caspase-dependent signaling pathways.

## Introduction

Okino *et al* (1) introduced the concept of electrochemotherapy (ECT), on the basis of electroporation, in 1992. Electric pulses with a long length (ms–μs) have been shown to induce a transient rearrangement of the lipid bilayer of cells and form aqueous channels in the cell membrane, this is termed electroporation (2). Hofmann *et al* (3) and Dev *et al* (4) applied electrochemotherapy in conjunction with bleomycin, to treat cancers, this strategy significantly reduced the side effects of the drug. Electric pulses with a short duration (ns–ps) have been shown to mainly affect intracellular organelles and cause apoptosis, as well as cytoskeletal, nuclear membrane and DNA damage, whilst maintaining an intact plasma membrane (5–7). According to the time-domain theory (8), psPEF has a wealth of ultra-wideband (UWB) spectrum (almost from direct current up to GHz), therefore it has a higher time and spatial resolution, with little distortion of the signal. If psPEF may be used to target deeper target lesions, without harming the normal tissues, a non-invasive treatment for tumors may be achieved (9). Mitochondrial transmembrane potential has a crucial role in apoptosis. A lower mitochondrial membrane potential induces the release of cytochrome C and activates the caspase family, which ultimately leads to apoptosis (10).

Previous results have indicated that microsecond PEF (μsPEF) and nanosecond PEF (nsPEF) may induce apoptosis of SKOV3 and HeLa cells, through the mitochondrial and endoplasmic reticulum pathways (11–15). The aim of the present study was to evaluate the efficacy of psPEF on a cervical cancer xenograft. A cervical cancer xenograft was generated in nude mice as a model, and the effectiveness of certain psPEF parameters on the tumor was investigated *in vivo*.

## Materials and methods

### Chemicals and reagents

RPMI-1640 media and fetal bovine serum (FBS) were purchased from Gibco Life Technologies (Carlsbad, CA, USA); the Terminal Deoxynucleotidyl-transferase-mediated dUTP Nick End Labeling (TUNEL) Detection kit was obtained from Roche Diagnostics (Basel, Switzerland). The Mitochondria Isolation kit, Mitochondrial Membrane Potential Assay kit with JC-1, and Caspase-3, -8 and -9 Activity Assay kits were purchased from Beyotime Institute of Biotechnology (Haimen, China). A Bradford Protein Assay kit was purchased from Comwin Biotech Co., Ltd. (Beijing, China), and the Caspase-12 Quantification kit was purchased from Genmed Scientifics, Inc., USA (Shanghai, China).

### Cells

The HeLa human cervical cancer cells (Cell bank of Committee on Type Culture Collection of Chinese Academy of Sciences, Wuhan, China) were cultured in RPMI-1640, supplemented with 10% FBS, 100 U/ml penicillin and 100 μg/ml streptomycin, at 37°C and in an atmosphere containing 5% CO_2_. The cells were digested with trypsin, and 2×10^7^ cells/ml were prepared for subcutaneous injection.

### Tumor models

The female nude mice were obtained from the Laboratory Animal Center of Chongqing Medical University, (Chongqing, China), aged six weeks. The mice were fed under specific pathogen-free conditions, and were randomly divided into four groups (six mice per group). A HeLa cell/Matrigel mixture of 0.2 ml was injected into the dorsal subcutis of the nude mice. The psPEF treatment was performed once the xenograft had reached a diameter of 0.8–1.0 cm (~2 weeks). Ethical approval of animal procedures was obtained from the Second Affiliated Hospital of Chongqing Medical University.

### Exposure to electrical pulses

**Figure 1:**
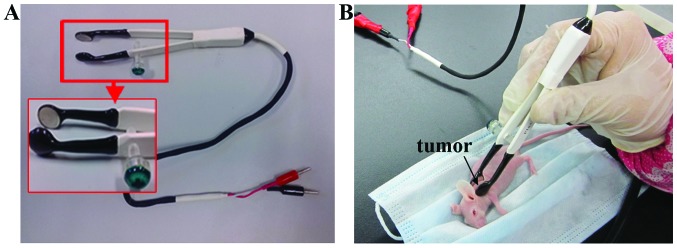
(A) Image of the tweezer-electrodes. The shape of the tweezer-electrodes that came into contact with the tumor was flat. (B) The tumor generated in the nude mouse model was compressed between the tweezer-electrodes, following anaesthetization.

Following anesthesia by intraperitoneal injection of 0.7 ml 10% chloral hydrate, the tumor was placed closely and non-invasively between the tweezer-electrodes (Fig. 1), and exposed to 2,000 pulses, at a 3 Hz frequency for 800 psec, with 120–140 kV/cm strength. The electric field amplitude and pulse width were monitored throughout the procedures using a DP04054 oscilloscope (Tektronix, Inc., Beaverton, OR, USA). The control mice were not treated, and the tumor tissues were harvested once the tumor had reached a diameter of 0.8–1.0 cm. In the experimental 6 h group, the mice were sacrificed and the xenografts were harvested 6 h following electrical pulse exposure. In the 12 h group, the nude mice were sacrificed by cervical dislocation 12 h following electrical pulse exposure and the tumors were harvested. In the 24 h group, the nude mice were sacrificed and the tumors were harvested 24 h following electrical pulse exposure.

### Ultrastructural study

The tumors were fixed in cacodylate-buffered 1% osmium tetroxide, dehydrated, and embedded in Epon 812 for ultra-thin sectioning. The tissue sections were stained with uranyl acetate and lead citrate and observed under a transmission electron microscope (TEM; H-7500; Hitachi Ltd., Tokyo, Japan).

### TUNEL assay

The tissue sections were incubated in terminal deoxynucleotidyly-transferase (TdT) buffer and TdT reaction solution mixture. Briefly, the sections were soaked in 2X saline sodium citrate solution for 10 min, in order to terminate the reaction. The sections were then incubated in Chain-avidin-marked horseradish peroxidase enzyme for 30 min, and stained with 0.04% DAB for 5–10 min and hematoxylin for 3–5 min. The sections were processed using a TUNEL Detection kit, according to the manufacturer’s instructions. The TUNEL-positive cells, per field, were counted in 10 random fields, and the counts were averaged. Apoptotic index = (number of apoptotic cells/number of all tumor cells) × 100%.

### Laser scanning confocal microscopy (LSCM) evaluation of mitochondrial transmembrane potential

The tumor tissues were homogenized in Mitochondria Isolation Reagent A and the homogenates were centrifuged at 600 × g for 5 min, at 4°C. The sediment was then stained using the ΔΨm-specific probe JC-1 for 5–10 min. The samples were visualized using LSCM (TCS-SP2; Leica, Wetzlar, Germany) with fluorescein isothiocyanate (green) and rhodamine isothiocyanate (red) channels. Red fluorescence was considered to represent a higher mitochondrial membrane potential, whereas green fluorescence was considered to represent a lower mitochondrial membrane potential. The two fluorescent images (green and red) were analyzed using confocal software (PCS-SP2 confocal laser fluorescence semi-quantitative analysis software; Leica) which allowed for the quantification of the intensity of green and red fluorescence.

### Caspase-3, -8 and -9 activity assay

The activity of caspase-3, -8 and -9 was measured using commercially available kits. Briefly, the tumor samples were homogenized in lysis buffer and the homogenates were centrifuged at 16,000 × g, for 15 min at 4°C. The supernatants were collected and the protein concentrations were determined using the Bradford Protein Assay. Caspase-3, -8, and -9 activity was measured using substrate peptides Ac-DEVD-pNA. The absorbance was measured at a wavelength of 405 nm, using an EXL 800 Universal Microplate Reader (BioTek Instruments Inc., Winooski, VT, USA).

### Caspase-12 activity assay

The frozen tumor was ground to a powder and transferred to a tube containing 150 μl lysis buffer. The tissue was homogenized and centrifuged at 16 × g for 10–15 min, at 4°C. The supernatant was transferred to a new tube and incubated for 90 min. The absorbance was measured at a wavelength of 405 nm using the EXL 800 Universal Microplate Reader.

### Inhibition rate

The nude mice were sacrificed and the tumor samples were harvested. The inhibitory rate was subsequently calculated on the seventh day following psPEF treatment. Inhibition rate (%) = (1 − average weight of the tumor in the treated group/average weight of tumor in the untreated group) × 100%.

### Statistical analysis

All of the data were processed using the statistical software SPSS version 17.0 (SPSS Inc., Chicago, IL, USA). The student’s t-test, a one-way analysis of variance and a χ^2^ test were used to determine the statistical significance of the differences between the groups. A P<0.05 was considered to indicate a statistically significant difference.

## Results

### Histology

**Figure 2:**
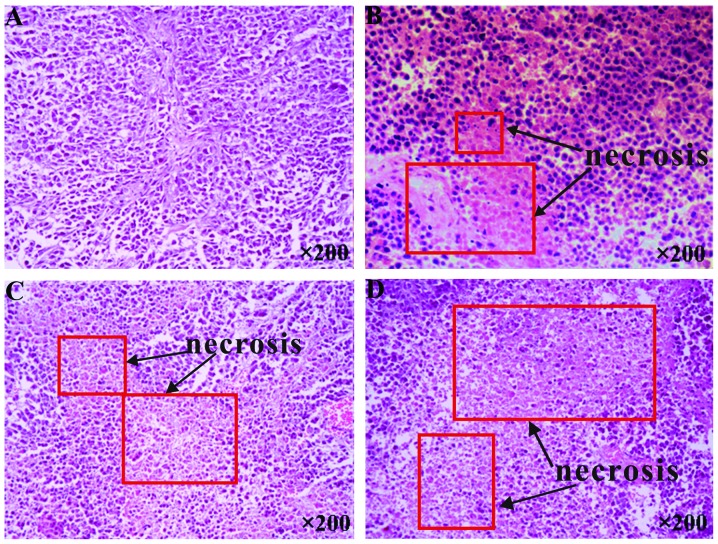
Necrosis of the human cervical carcinoma in a nude mice xenograft model, as captured under an optical microscope. The tumor was removed from the mice sacrificed at 6, 12 and 24 h following picosecond pulsed electric fields (psPEF) treatment and the control mice. (A) Controls, not treated by psPEF; (B) 6 h, (C) 12 h and (D) 24 h, following exposure to psPEF treatment. Light staining and pink homogeneous regions represent necrotic tumor cells. Normal tumor cells exhibit a dark staining and a clear outline. A significant increase in necrosis was observed in the treated groups, which gradually increased with the processing time, and was most notable in the 24 h group. Magnification, ×200.

**Figure 3:**
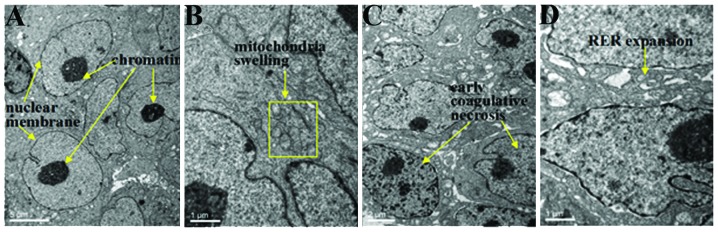

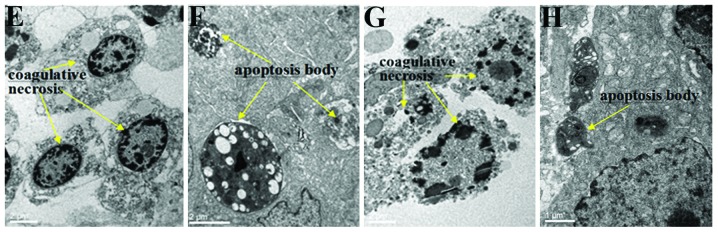
Ultrastructure of the human cervical carcinoma in the nude mice xenograft model under transmission electron microscopy. (A and B) Controls, not treated with picosecond pulsed electric fields (psPEF), and the cells at (C and D) 6 h, (E and F) 12 h and (G and H) 24 h following exposure to psPEF treatment. The cells of the control group were intact, with (A) well distributed chromatin, a clear nuclear membrane, and (B) mild mitochondrial swelling. However, the cells of the 6 h group demonstrated (C) early coagulative necrosis with (D) rough endoplasmic reticulum expansion. The 12 h group had (E) notable coagulative necrosis but the cell outline remained visible, a segmentally fractured plasma membrane and (F) numerous apoptotic bodies. The 24 h group had (G) severe coagulative necrosis, in which the cell outline disappeared, and there were (H) few apoptotic bodies. Following the psPEF treatment, apoptotic bodies appeared, which were markedly evident in the 12 h group, and necrosis of the cells increased which was most notable in the 24 h group. Magnification: A, ×4,000; B,:x15,000; C, ×7,000; D, ×15,000; E,:x15,000; F, ×7,000; G, ×7,000; H, ×15000.

In the pathological examinations, necrosis was not observed to be present in the control tumors, whereas psPEF treatment led to cell necrosis (Fig. 2B), which was most notable in the 24 h group (Fig. 2D). The TEM demonstrated that the control tumors exhibited mild mitochondrial swelling (Fig. 3A and B). The tumors that underwent psPEF treatment showed early coagulative necrosis in the 6 h group (Fig. 3C and D); notable coagulative necrosis (Fig. 3E) and numerous apoptotic bodies (Fig. 3F) in the 12 h group; and severe coagulative necrosis (Fig. 3G) and few apoptotic bodies (Fig. 3H) in the 24 h group.

### Rate of apoptosis

**Figure 4:**
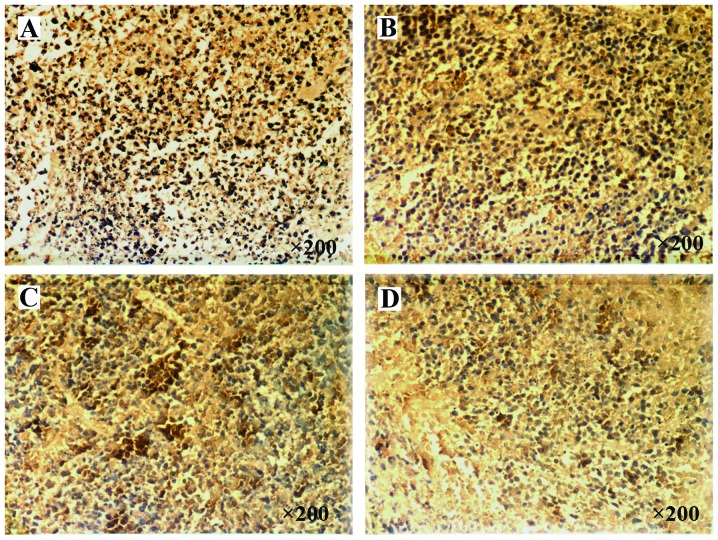

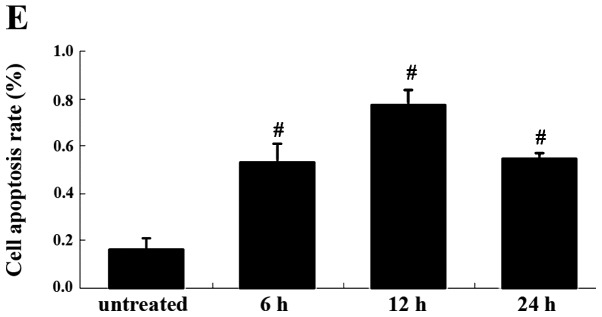
Apoptotic rate of the cells from human cervical carcinoma in a nude mice xenograft model. A brown-yellow cell nucleus represented an apoptotic cell. (A) Control group; groups at (B) 6 h, (C) 12 h and (D) 24 h, following exposure to picosecond pulsed electric fields (psPEF) treatment. Magnification, ×200 (E) Histogram plotted according to the data in A–D. The data are presented as the means ± standard deviation of three separate experiments. ^#^P<0.01; comparison between two neighboring groups. Following the psPEF treatment, the rate of apoptosis increased, which was most notable in the 12 h group.

There was an increased rate of apoptosis in all of the treatment groups, as compared with the control group (P<0.01; Fig. 4E). The apoptotic rate of the 12 h group was significantly higher, as compared with the other treatment groups (P<0.01; Fig. 4E). However, there were no statistical differences between the 6 and 24 h groups.

### Mitochondrial transmembrane potential

**Figure 5:**
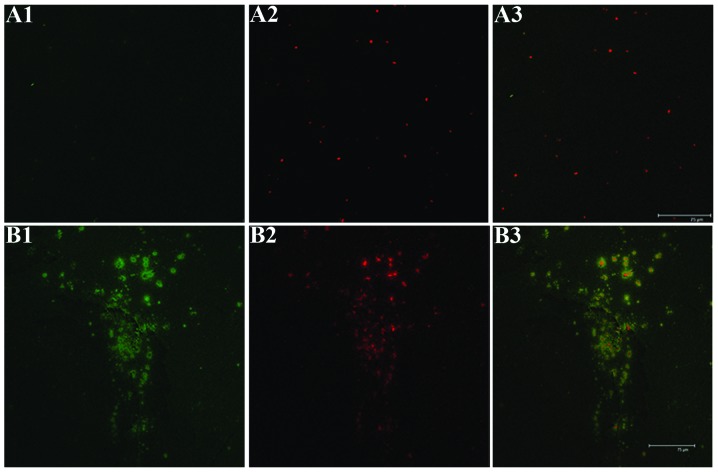

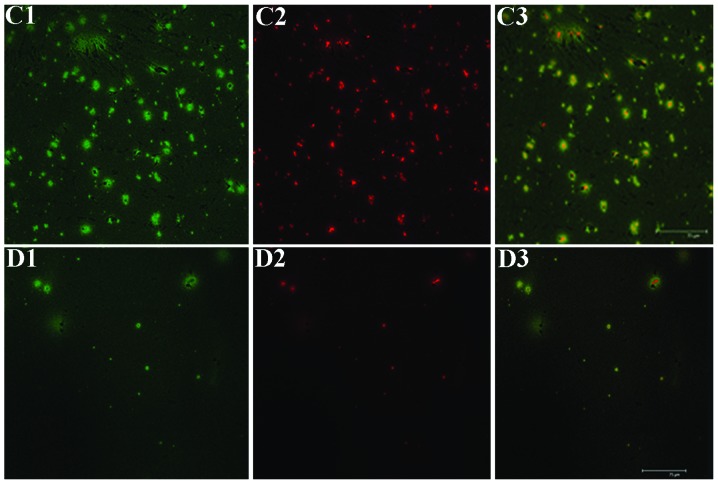
Mitochondrial transmembrane potential changes of the human cervical carcinoma in a nude mice xenograft model. (A) Control group; groups at (B) 6 h, (C) 12 h and (D) 24 h following picosecond pulsed electric fields (psPEF) treatment. Magnification, ×400. The green and red fluorescence represents the declined mitochondrial membrane potential of early apoptotic cells, and the normal potential of cells, respectively. The red fluorescence weakened and the green fluorescence increased in the 6 and 12 h groups, as compared with the control group. However, the two fluorescent signals were weak in the 24 h group. 1, green fluorescence only; 2, red fluorescence only; 3, merge.

When stained with the mitochondria-specific probes JC-1, the mitochondria of tumor cells may emit red and green fluorescence. The experimental 6 and 12 h groups presented a reduced red fluorescence and increased green fluorescence, as compared with the untreated controls (Fig. 5A–C). However, the experimental 24 h group presented weak red and green fluorescence, as compared with the 6 and 12 h groups (Fig. 5D).

### Caspase-3, -8, -9 and -12 activity

**Figure 6:**
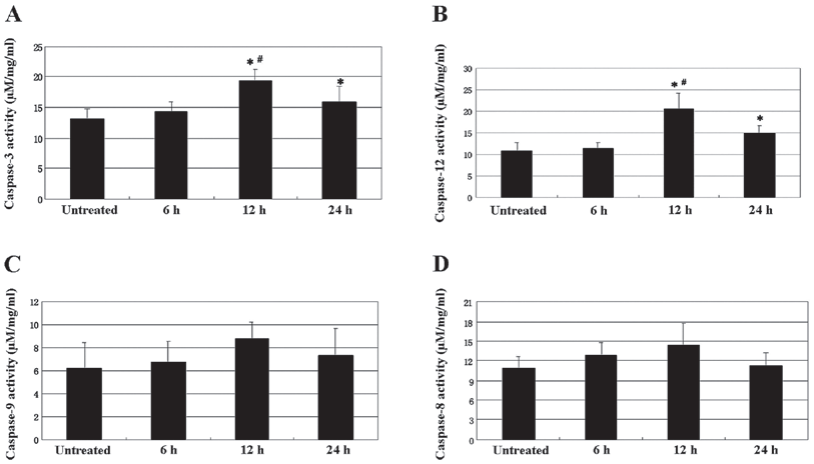
The activity of the activated caspases-3, 8, 9, 12 in the human cervical carcinoma from a nude mice xenograft model. Histograms for (A) caspase-3 (B) caspase-12, (C) caspase-9, (D) and caspase-8 activity. The data are presented as the means ± standard deviation of three separate experiments. ^*^P<0.05 and ^#^P<0.01. Caspase-3 and 12 activity was markedly higher in the 12 h group, as compared with the other groups. There was no significant difference in the activity of (C and D) caspases-8 and 9 activity between the control and the experimental groups.

The activity of caspase-3 and caspase-12 was increased in the tumors, following exposure to psPEF. Caspase-3 activity in the various groups had the following hierarchical pattern: 12 h>24 h>6 h=control group (P<0.05; Fig. 6A). Caspase-12 activity in the various groups had the following hierarchical pattern: 12 h>24 h>6 h=control group (P<0.01; Fig. 6B). However, caspase-8 and -9 activity were not affected by exposure to psPEF (Fig. 6C and D).

### Inhibition rate

The rate of inhibition was 9.11% at the seventh day following exposure to psPEF. This demonstrated that, to some extent, picosecond pulsed electric fields are able to inhibit tumor growth in a xenograft tumor model of cervical cancer.

## Discussion

The results of the present study suggest that psPEF may induce cellular apoptosis of a cervical cancer xenograft, by activation of the endoplasmic reticulum stress signaling pathway.

For the purpose of preserving fertility, an effective and non-invasive therapy for cervical carcinoma is required. PEF is a novel technique. psPEF may be transferred to non-invasive target deep tissue (8,16), thus resulting in non-invasive treatment. Previous studies have demonstrated that psPEF is an efficient apoptosis-inducing agent in HeLa cells, through the endoplasmic reticulum stress and caspase-dependent signaling pathways (15). However, there are differences between cellular *in vitro* and *in vivo* experiments. Cell activity is affected by humoral regulation and other factors *in vivo*. The presence of both cervical cancer and normal tissue may have an effect on the efficacy of psPEF *in vivo*. Therefore, a study determining the effects of psPEF on a cervical cancer xenograft was required, which is more relevant for clinical applications. The present study demonstrated that psPEF is effective on cervical carcinoma *in vivo*. psPEF led to tumor cell death in a cervical carcinoma xenograft, which gradually increased with prolonged exposure, during which there was significant tumor cell apoptosis. These results suggest that apoptosis has an important role in tumor cell death. Also, LSCM showed that the mitochondrial membrane potential of the tumor decreased following psPEF treatment, and the number of mitochondria in the 24 h treatment group was reduced, as compared with the 12 h group. These results indicate that some apoptotic tumor cells may die and be subsequently cleared away. Furthermore, the tumor inhibition rate suggested that psPEF could effectively inhibit tumor growth *in vivo.*

The alterations to cellular protein activation associated with psPEF stimulation may provide valuable information to determine the occurrence of stress, and also to help elucidate the mechanisms of action of pulses on cervical carcinoma *in vivo*. The present study suggested that psPEF induced tumor cell apoptosis through the endoplasmic reticulum stress and caspase-dependent signaling pathways.

In conclusion, the present study demonstrated the efficacy of psPEF on cervical carcinoma *in vivo*. These findings may be used to develop a non-invasive therapeutic strategy. A future study may include a larger sample size and modified psPEF parameters. Additional studies on the safety of psPEF *in vivo* are currently underway.
